# The effect of Rebozo technique on perceived labour pain and childbirth experience: A randomized controlled trial

**DOI:** 10.1097/MD.0000000000039346

**Published:** 2024-08-30

**Authors:** Özden Tandoğan, Ümran Oskay

**Affiliations:** a Faculty of Health Sciences, Department of Nursing, Istanbul Arel University, Istanbul, Turkey; b Faculty of Nursing Department of Gynecology and Diseases Nursing, Istanbul University-Cerrahpaşa Florence Nightingale, Istanbul, Turkey.

**Keywords:** birth, childbirth experience, labor pain, rebozo technique

## Abstract

**Background:**

Pain, stress, and anxiety experienced during childbirth can have detrimental effects on labor and delivery. The rebozo technique is an ancestral method used to minimize pain and enhance relief during gestation. This study aimed to investigate the effects of the rebozo technique on the birth process and its probable benefits on the birth experience.

**Methods:**

This survey was conducted from January to May 2021 in a randomized and controlled manner. A total of 113 pregnant women with their first children were surveyed. Women between 37 and 41 weeks of gestation without complications who were admitted to the delivery room with a cervical dilation of 4 cm or more were chosen as participants. In the Rebozo group, subjects were randomly selected by trained personnel to apply the standardized method, while the control group received a relaxing massage. Cervical dilation, fetal position, contraction patterns, and measures related to the birth experience were key indicators.

**Results:**

Women in the rebozo group had lower pain levels during birth and greater birth satisfaction. Mean cervical dilation in the latent phase was 5.61 cm in the rebozo group and 5.71 cm in the control group. In the active phase, cervical dilatation was 6.03 cm in the rebozo group and 6.68 cm in the control group, and this difference was statistically significant (*P* < .001). In the transition phase, the birth time was 46.29 minutes in the rebozo group and 68.71 minutes in the control group (*P* = <.007**). In the total birth experience score, the rebozo group received an average of 68.52 points, while the control group received 51.58 points (*P* < .001).

**Conclusion:**

This research has established that the use of the rebozo technique throughout labor helps enhance her feelings about being pregnant, as well as heightening fulfillment with delivery.

## 1. Introduction

It is important to note that the birth process is a major milestone in women’s lives with many physical and emotional challenges. Pain, stress, and anxiety experienced by women during this process can negatively affect their birth experience.^[1]^ Therefore, other methods to help women relax their bodies and reduce pain during delivery are under investigation. Over the years, the rebozo technique has become popular as a method for childbirth. Rebozo technique is a traditional Mexican method that has been increasingly used in pregnancy and delivery as a massage and support technique. This involves the use of rebozo, a special long shawl made of cotton fabric that is commonly worn in Mexico. By making different movements throughout her body, rebozo technique helps mothers relieve labor pain while relaxing them by tightly hugging their bodies during birth.^[2]^

Research supports the rebozo technique as an intervention for labor pain. Numerous studies have documented that the rebozo technique decreases pain during labor, making it a more comfortable experience for women. Women who use rebozo technique experience less pain and participate in a positive birthing process.^[3,4]^ However, it should be understood that the effect of this technique on the birth experience is not only limited to relieve pain. As demonstrated by the application of rebozo, mothers were more relaxed, less anxious, and positively prepared for delivery. Moreover, with the help of the rebozo method, mothers have been said to feel extra control during the time of giving, thereby aiding them in adopting a natural position. They also offer support to mothers during birth. Fathers or doulas can actively participate in rebozo, which can make birth positive.^[2]^ Therefore, emotional support and empowerment can be offered to mothers, resulting in an improved childbirth experience.

An effective method known as the rebozo technique helps women relieve pain, relax, and have a positive birth experience.^[3]^ In general, there is evidence that the use of rebozo during labor has various benefits. Therefore, when this method is used during childbirth, the process becomes easier for women.^[2–5]^ Regarding the effects of the rebozo technique on the birth experience itself, this approach may enable women to enjoy their births more. This can help them go through labor without much stress and anxiety by supporting their partners who are going through this phase. Mother-infant rebozo care increases the comfort of both mothers and children, thereby promoting postpartum bonding. As such, proceeding with these methods and supporting their utilization in giving birth would enhance natural childbirths among females while contributing to better health and happiness levels.^[6]^ However, further studies should be conducted on the benefits of using the rebozo technique to determine whether these claims are true. Although this system appears to be traditional, additional scientific trials are required to determine its impact. Competents must therefore employ these techniques. Clearly, this technique may be useful in reducing labor pain and increasing birth experience. However, there must be further research on it on which should be taken up by specialists. The fact remains that The rebozo technique may help reduce labor pain and assist in smooth delivery. However, further research needs to be conducted by experts regarding this issue. The use of the rebozo technique as an alternative means of comforting pregnant women during labor should be considered. Understanding why certain things happen at birth will lead to a complete understanding of both their importance and effects at different levels, including the physical aspects related directly to medical interventions.^[1]^ Suggestions will be made regarding how clinical practices can incorporate the rebozo technique into the birth process.

## 2. Methods

This randomized controlled study was conducted between January and May 2021. This study provided a detailed description of the subjects, substances, and tools used. Participants were selected from among women with a gestation period between 37 and 41 weeks, who had their first pregnancy with a single fetus, did not have any risk factors during pregnancy, had a fetus in a longitudinal lie with vertex presentation, had a body mass index (BMI) below 30 at the time of delivery, and were admitted to the labor room at 4 cm. In total, 113 primiparous pregnant women were recruited for this study. Age, sex, race/ethnicity, and financial status of participants were recorded as part of the demographic data collection process. The inclusion standards were established based on the objectives of this study. The exclusion criteria were as follows: women who opted for cesarean section due to failed labor trial or those who received any form of medical intervention, non-Turkish speaking individuals, or those who did not work but wishing to quit work.

The preparation of the study materials and the selection of individuals to participate in the study were performed with great care, ensuring that they were done meticulously. The research material was well-prepared, and it can be concluded that the objectives and hypotheses were met by the people involved in this study. After randomly selecting participants, they were assigned to either the rebozo or control group. Randomization ensured that the participants were evenly distributed into 2 groups. Researchers were required to know who these individuals belonged to, because of the nature of the study, whereas those responsible for the data remained anonymous. All pregnant women assigned to the experimental or control group stayed in separate rooms where communication between them was not allowed.

The rebozo technique has been applied in a standardized manner by practitioners with sufficient training. Thus, the correct and consistent application of this technique is guaranteed. The control group received relaxing massage as a physical intervention. This enables a more accurate assessment of the effects of the rebozo technique.

Scales with proven validity and reliability were used as data collection tools. These scales have been adapted and validated in Turkish society. The measurement and calculation methods have been explained in detail. Information regarding the data collection procedures, timing, and frequency of the measurements was detailed. Measurement tools and data collection methods were carefully selected and validated.

**Figure 1.**
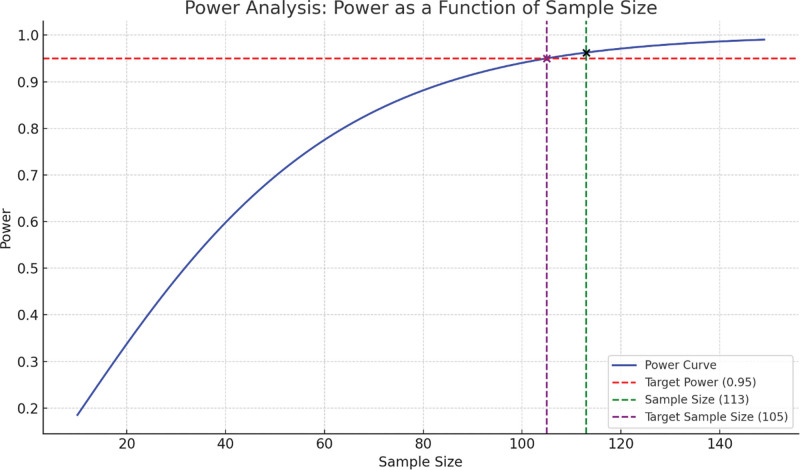
Power analysis results.

The statistical analysis methods have been explained in detail. The software and tools used to analyze the data were specified, and the analysis process was detailed. Missing data were handled and multiple comparisons were performed. Based on calculations using the G*Power program, the effect size was 0.93, and the minimum sample size required to provide 95% power was calculated as at least 58 participants (rebozo group) and 55 participants (control group) for each group (Fig. 1).

### 2.1. Ethical review, methods section

This study was conducted in accordance with the ethical standards of the Declaration of Helsinki. This study was approved by the Istanbul University-Cerrahpaşa Faculty of Medicine Noninvasive Clinical Research Ethics Committee (date: December 07, 2020/Decision No 59491012-604.01). Informed consent was obtained from all participants.

**Figure 2.**
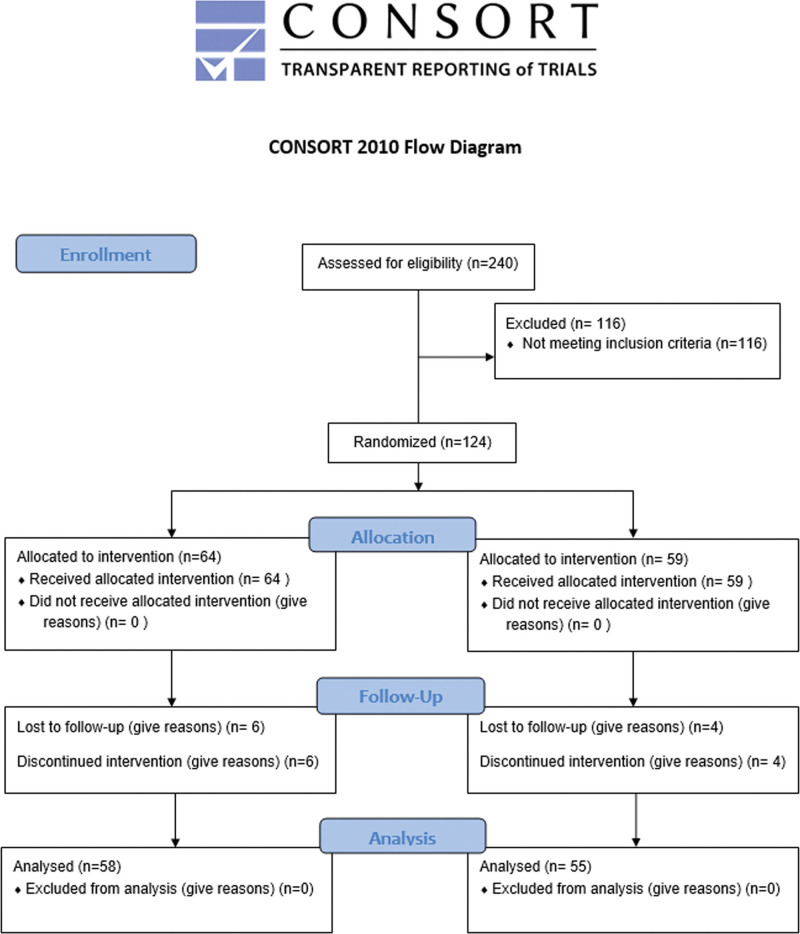
Study design and CONSORT flow diagram.

The study design and research steps were based on the CONSORT guidelines (Fig. 2).

**Figure 3.**
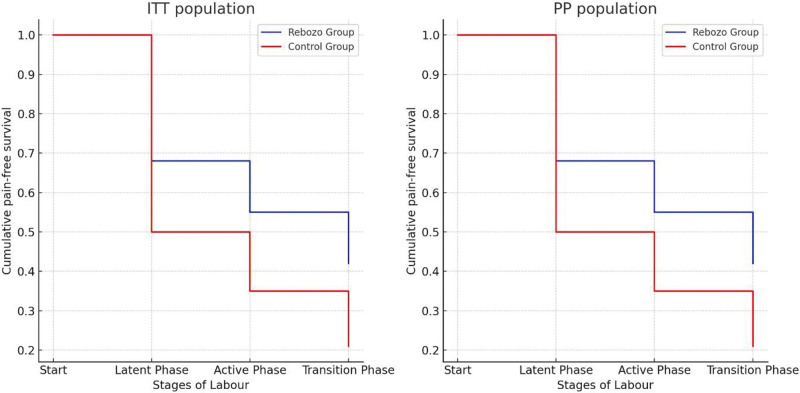
Kaplan–Meier curves for ITT and PP populations. ITT = intention-to-treat, PP = per protocol.

Intention-to-treat analysis was performed to compare pain levels in rebozo and control groups at different stages of labor (latent phase, active phase, transition phase). Cumulative pain-free survival rates (PP Population: Per Protocol) were calculated using Kaplan–Meier curves. Pain levels (visual analog scale [VAS] scores) were graded from 0 to 10 and calculated for each stage (Fig. 3).

## 3. Results

This study aimed to investigate the effects of the rebozo technique on pain management during labor and birth. Demographic characteristics, cervical dilation, fetal position, contraction characteristics, and birth experience were evaluated. The aim of these findings was to establish the efficiency of the rebozo technique by comparing rebozo and control groups.

There were no statistically significant differences between the rebozo and control groups in terms of demographic characteristics such as age, education period, duration, and employment status among the study participants. While the average age of the women in the rebozo group was 23.27 ± 4.50, the average age of the women in the control group was 22.70 ± 3.70 (*P* = .441). The average duration of education in both groups was 7.90 years and there was no statistically significant difference (*P* = .932). The average duration of marriage was found to be 1.50 ± 0.80 years in the rebozo group and 2.00 ± 1.60 years in the control group, and this difference is not statistically significant (*P* = .426). In terms of employment status, 19.0% (11 people) of the women in the rebozo group were working, while 9.1% (5 people) of the control group were working (*P* = .473). In terms of pregnancy planning status, while 86.2% (50) of the women in the rebozo group planned their pregnancy, this rate was 78.2% (43) in the control group (*P* = .508). The proportion of women who underwent regular prenatal checkups was 84.5% (49 women) in the rebozo group and 100% (55 women) in the control group, and the difference was statistically significant (*P* = .053). In terms of gestational age, women in the rebozo group had an average gestational age of 38.60 ± 1.40 weeks, while women in the control group had an average gestational age of 39.50 ± 1.20 weeks, and this difference was found to be statistically significant (*P* = .007**). The averages of the pre-pregnancy BMI were similar for both groups, 22.27 ± 4.08 in the rebozo group and 22.62 ± 4.59 in the control group (*P* = .994). Similarly, BMI values during pregnancy were found to be 27.86 ± 4.42 in the rebozo group and 28.05 ± 4.43 in the control group, and no significant differences were observed (*P* = .844) (Table 1).

**Table 1:** Demographic information of participants.

Variable	Rebozo group (n = 58)	Control group (n = 55)	*P* value
Age (mean ± SD)	23.27 ± 4.50	22.70 ± 3.70	.441
Duration of education (mean ± SD)	7.90 ± 4.50	7.90 ± 4.00	.932
Duration of marriage (mean ± SD)	1.50 ± 0.80	2.00 ± 1.60	.426
Employment status, n (%)			
Employed	11 (19.0)	5 (9.1)	.473
Unemployed	47 (81.0)	50 (90.9)	
Planned pregnancy, n (%)			
Yes	50 (86.2)	43 (78.2)	.508
No	8 (13.8)	12 (21.8)	
Regular antenatal control, n (%)			
Yes	49 (84.5)	55 (100.0)	.053
No	9 (15.5)	0 (0.0)	
Number of antenatal controls, n (%)			
1–4	9 (15.5)	3 (5.5)	.425
4 and more	49 (84.5)	52 (94.5)	
Gestation week (mean ± SD)	38.60 ± 1.40	39.50 ± 1.20	.007**
BMI before pregnancy (kg/m^2^) (mean ± SD)	22.27 ± 4.08	22.62 ± 4.59	.994
BMI during pregnancy (kg/m^2^) (mean ± SD)	27.86 ± 4.42	28.05 ± 4.43	.844

BMI = body mass index.

** Significant at *P* < 0.05.

**Figure 4.**
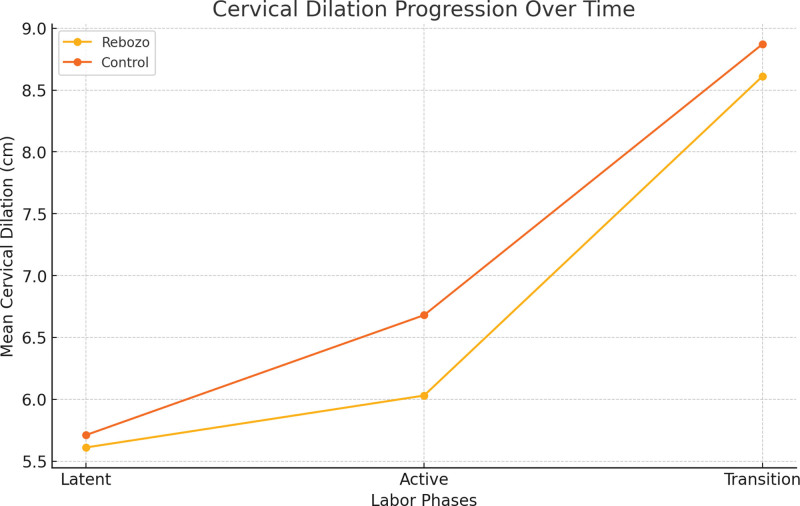
Labour-related characteristics comparison.

The research findings showed that the rebozo and control groups had different cervical dilatations, fetal positions, and contraction characteristics. The mean cervical dilation in the latent phase was 5.61 cm in the rebozo group, and 5.71 cm in the control group, with no significant difference. Furthermore, the mean cervical dilation in the active phase was 6.03 cm for the rebozo group versus 6.68 cm for controls with significant difference between the 2 groups as well (*P* < .001) The transition phase was found to be 8.61 cm in the rebozo group and 8.87 cm in control group, respectively. There were no statistically significant differences between the groups for fetal head level and fetal heart rate, although they differed numerically in several instances; for example, during the latent phase it was recorded as 2.90 for the rebozo, however, 2.94 between the controls; while rate varied from 2.55 and 2.65 respectively; Similarly during the latent phase, the fetal heart rate varied from 138.97 (rebozo) to 13,939 (control). The active phase showed an insignificant difference between the 2 groups; the average number of contractions per minute was recorded at “... in the rebozo…” of “... among controls” with no significant difference observed (*P* > .05). During the transition period, this value was equal to be equal to 3/19 seconds within which it increased to 3/90 units (*P* < .001). Contraction frequency, however, decreased from the duration of contractions to only, while its duration dropped from 179.68s (rebozo) compared with 187.1s (control) in the case of active intensity, respectively, at the transition stage where this time span increased up to 2029s against 16097s; however, these differences were significant only during the transition period of labor. The average intensity during the latent phase was 28.84 for the Rebozo group and 32.32% in the control group, which was significant. However, there was no difference in the contraction intensity between the active and transition phases between the groups. Regarding the length of labor, although there were no significant differences between the 2 groups in terms of time of birth during the latent phase, different observations were made, with remarkable dissimilarities placed on the active stage as well as transition phases between the rebozo technique and conventional methods. Birth time was recorded in the transition period whereas it took 46.29 minutes for Rebozo group and control group reported 68.71 minutes, respectively. The duration of the second stage is significantly different from that of someone spending only 15.9 seconds while another is occupied for more than half an hour (31/58) (*P* < .05). Thus, these results indicate that the rebozo technique is sufficiently efficient during pregnancy and has some positive effects, especially in terms of pain relief and labor hours (Table 2; Fig. 4).

**Table 2:** Labour-related characteristics of pregnant women.

Characteristics		Rebozo group (n = 58)	Control group (n = 55)	*P* value
Cervical effacement (%)	Latent phase (4–5 cm)	61.61 ± 8.98	60.97 ± 7.90	.649
	Active phase (6–8 cm)	69.68 ± 8.36	64.58 ± 13.31	.110
	Transition phase (8–10 cm)	65.81 ± 26.55	65.55 ± 26.45	.963
Cervical dilation (cm)	Latent phase (4–5 cm)	5.61 ± 0.50	5.71 ± 0.46	.425
	Active phase (6–8 cm)	6.03 ± 0.18	6.68 ± 0.60	<.001**
	Transition phase (8–10 cm)	8.61 ± 0.8	8.87 ± 0.62	.168
Fetal head station	Latent phase (4–5 cm)	2.90 ± 0.30	2.94 ± 0.25	.644
	Active phase (6–8 cm)	2.55 ± 0.57	2.65 ± 0.61	.369
	Transition phase (8–10 cm)	1.90 ± 0.94	1.61 ± 0.76	.094
Fetal heart rate	Latent phase (4–5 cm)	138.97 ± 9.17	139.39 ± 9.04	.351
	Active phase (6–8 cm)	141.00 ± 6.94	140.77 ± 6.13	.750
	Transition phase (8–10 cm)	132.03 ± 10.48	131.77 ± 13.45	.657
Number of contractions (in 10 min)	Latent phase (4–5 cm)	2.42 ± 0.67	2.35 ± 0.84	.634
	Active phase (6–8 cm)	3.45 ± 0.2	3.48 ± 0.96	.898
	Transition phase (8–10 cm)	3.19 ± 0.87	3.90 ± 0.79	<.001**
Contraction frequency time (s)	Latent phase (4–5 cm)	262.9 ± 103.25	291.94 ± 132.35	.419
	Active phase (6–8 cm)	179.68 ± 34.69	187.1 ± 58.26	.898
	Transition phase (8–10 cm)	202.9 ± 59.17	160.97 ± 38.76	<.001**
Contraction severity	Latent phase (4–5 cm)	28.84 ± 4.39	32.32 ± 5.72	.013*
	Active phase (6–8 cm)	43.1 ± 11.19	48.26 ± 11.43	.064
	Transition phase (8–10 cm)	71.52 ± 9.55	76.06 ± 12.79	.098
Phase duration (min)	Latent phase (4–5 cm)	118.06 ± 52.24	118.71 ± 70.56	.783
	Active phase (6–8 cm)	76.52 ± 46.74	83.71 ± 48.89	.528
	Transition phase (8–10 cm)	46.29 ± 40.76	68.71 ± 36.51	<.007**
	Duration of the second phase (min)	15.97 ± 19.17	31.58 ± 19.10	<.001**

*P < 0.05; **P < 0.01.

**Figure 5.**
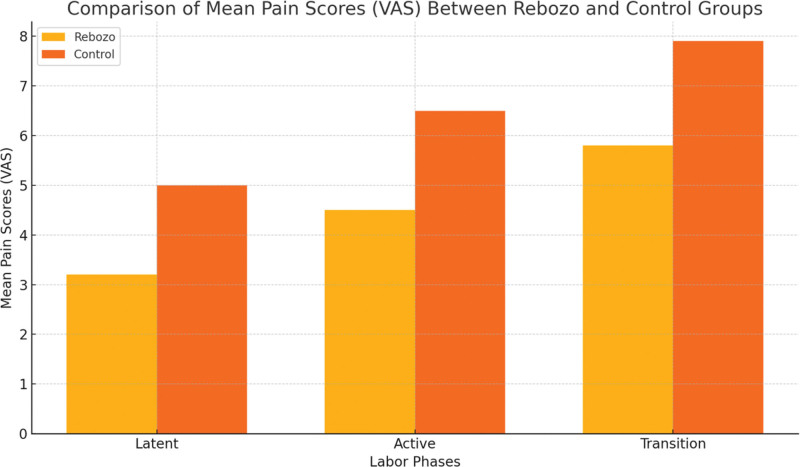
Comparison.

Research findings have shown that rebozo and control groups differ significantly in terms of subdimensions and total scores on the birth experience scale. The average participation score of those involved in decision-making processes was 9.84 for the rebozo group and 6.58 for the control group; while the birth process subdimension scored 23.65 points among the former, it was recorded as 17.97 in relation to the latter. Among other things: professional help and support received an average rating of 15.45 (rebozo) versus 11.90 (control); safety perception/memory received an average score of 19.58 (rebozo) compared to 15.13 (control). However, based on the total birth experience score, there were records such that while rebozo averaged about 68.52 points controls only managed to get around half this number, that is, approximately 51.58 points each, which was statistically significant at the *P* < .001 level. In addition, VAS pain ratings showed that all 3 stages (latent, active, and transition phases) had lower measurements when performed using the rebozo method than when performed without it during labor pain measurement using the VAS. In the latent stage, pain scores for the rebozo group were measured as an average of 3.2 for the rebozo group, while the control group received a mean value equal to 5 points. Similarly, during the active phase, there was a difference between the rebozo and non-rebozo groups, where they scored 4 points 5 and 6 points 5, respectively, and during the transition period, the 7-point 9 rating was given by controls against 5 points, 8 scored among those who underwent the rebozo technique. Therefore, this evidence implies that this particular method improves the general labor experience satisfaction levels (Table 3; Fig. 5).

**Table 3:** Childbirth experience scale scores.

Sub-dimension	Rebozo group (n = 58)	Control group (n = 55)	*P* value	Effect size (η²)
Participation in decisions	9.84 ± 1.49	6.58 ± 1.88	<.001**	0.617
Labor process	23.65 ± 3.80	17.97 ± 4.05	<.001**	0.722
Professional help/support	15.45 ± 2.22	11.90 ± 3.88	<.001**	0.513
Detected safety/memories	19.58 ± 2.45	15.13 ± 3.47	<.001**	0.631
Childbirth experience total score	68.52 ± 6.55	51.58 ± 10.84	<.001	

** *P* < 0.05.

This study showed that the use of the rebozo technique significantly improved pain management during labor and the overall experience of birth. In the group that used the rebozo technique, there was a more controlled progression of cervical dilation, much lower intensity of labor pain, and higher levels of satisfaction with birth. These results indicate that non-pharmacological interventions, such as repositioning women during labor, may help them cope better with their contractions, thereby making it unnecessary for them to request drugs. The rebozo method is a safe way to ease discomfort during birth while also enhancing this natural process and promoting positive feelings about giving life. Therefore, it should be adopted widely within maternity units to increase satisfaction levels among women who choose it and reduce anxiety throughout delivery.

## 4. Discussion and conclusions

The factors that affect quality of life and health status are birth experience and pain management. Issues surrounding nature and management shape women’s experiences of giving birth, thus highlighting the need for further research in this field. Furthermore, Beigi et al^[7]^ underscored in their study what constitutes labor pain and related factors that adversely affect women’s birth experiences, while suggesting that better care should be given during its management. Susilawati^[8]^ also evaluated the efficacy of effleurage massage in alleviating labor pain and found that it was more effective than counterpressure massage in reducing labor pain. Suryani et al^[9]^ examined the impact of the rebozo technique in reducing labor, in addition to reducing potential risks for both the mother and baby by reducing labor duration; thus, finding possible complications can be reduced by using them on mothers during the delivery process. Finally, Hanum^[10]^ researched non-pharmacological methods used to relieve labor pain, stating that hot baths were particularly useful because they made birth processes less painful, even though these findings suggest that different strategies should be implemented to manage labor pain while enhancing the birthing experience.^[7–10]^

There were no significant differences in the demographic characteristics (age, education period, duration, and employment status) between the rebozo and control groups. Participants’ demographic characteristics were considered in the evaluation of pain relief methods during childbirth according to Wan Anita’s^[11]^ review of the system. Similarly, Amiri et al^[12]^ conducted a randomized control study that assessed whether distraction techniques reduced labor pain and anxiety as well as the role played by the demographic characteristics of the participants. Başgöl and Koç^[13]^ explored non–pharmacological ways to address pain, along with the influence that these approaches bring of demographics on their effectiveness. This study also revealed that both groups have similar demographic characteristics, thereby increasing reliability and validity. Therefore, it can be concluded that our results regarding the Rebozo technique are in line with those of other studies conducted in this area.^[11–13]^

This is the main event in women’s lives, and is an emotional and physical experience. Birth pain has always been an integral part of this process, and varies from one culture to another. In Turkish society, birth pain is widely regarded as something that cannot be endured; however, recent studies indicate that traditional methods, such as the rebozo technique, are helpful in reducing labor pain. Our study found that pregnant women who underwent the rebozo technique experienced reduced pain. This technique can be effective during both the active and latent phases; thus, decreasing the perception of pain and inducing feelings of relaxation among expectant mothers might help with labor pain. The fact that the average cervical dilation of women during the active phase was lower in the rebozo group than in the control group and the huge variations in terms of the number and intensity of contractions showed that this technique had positive effects on the birth process. Furthermore, among those in the transition phase, the rebozo group experienced shorter contractions and birth times than their counterparts; these discrepancies were statistically significant. Therefore, the rebozo technique could be viewed as an efficient option to handle pain associated with delivery processes, making it easy or comfortable for female patients.^[2,3,14,15]^

This study also examined the impact of the standing rebozo technique used in the second stage of labor. Another notable result was the decrease in the rates of the second stage of labor among pregnant women in the rebozo group. They help the baby navigate the birth canal by providing stability in the pelvic region. Table 3 presents the results of the comparison of the mean scores of the rebozo and control groups on the revised birth experience scale subdimensions and the total scores. The mean score on the participation of participants in the decision was 9. It was 84 in the rebozo group compared to 6 non-stem cell-related adverse events. 28 in the control group, respectively. Therefore, we included 83 and 58 patients in the experimental and control groups, respectively. The rebozo group received 23 beneficiaries in total according to data collected from different sources, as shown in the above table. It should also be noted that the birth process subdimension was significantly lower in the control group (17 out of 65 points). 97 points. Regarding the professional help and support sub-dimensions, the rebozo group had an average of 15. 45; while this value was 11.90 in the control group. The percentage of smokers in the control group was 90%. It is also important to note that the average score of the rebozo group was 19. Overall, in the subdimensions of security perception and memory, the respondents scored 15 and 58, respectively. Ten of the thirteen participants were assigned to the control group. The average total birth experience score was 68. 52 and 51, respectively, in the control group. 58 points. These differences were statistically significant (*P* < 0. 001). Compared with the control group, the SPB-rebozo group demonstrated significantly lower VAS pain scores in all 3 phases of the first and transition phases of the second stage of labor. The rebozo group reported an average pain score of 3. Two for the latent phase and 4. Five patients were in the active phase, and 5. The intervention group received 8 in the training phase of the study and 5 in the transition phase of the study, while the control group received 5 throughout the study. 0, 6. 5 and 7. 9 points, respectively.^[4,16]^

This research has some limitations, including the following: First, because the study was designed as a classic randomized controlled trial, participants and practitioners had to know to which group they belonged. This could have introduced a bias, which may have influenced the study outcomes. Second, variations in exposure or technique competency among the rebozo techniques used in this study may have affected the efficiency of the rebozo technique. Finally, the study design and sample size may restrict the generalizability of the findings. Thus, further investigations should be conducted to estimate the efficiency of this method in broader and more heterogeneous patient samples.^[16]^ Recognizing the possibility of positive results from the rebozo technique in the second stage of labor postulated by this study may be helpful for future research in obstetrics and the field of women’s health. According to the above studies, knowledge and implementation of the rebozo technique during childbirth can improve pregnant women’s experiences and satisfaction with delivery. This study also showed that the rebozo technique is a sufficient approach to help increase the quality of obstetric care and make childbirth less stressful for pregnant women. Given the positive outcomes found in this study, further research is required to determine their applicability in real-life settings. This technique should also be used in accordance with the guidelines established by relevant doctors. To enhance the generalization of the findings, studies should be conducted on a large population, including a large sample size. Other conditions should be addressed and further research should be conducted on the long-term consequences of the rebozo technique. Furthermore, courses and protocols of interest for health care workers must be provided to combine the use of the rebozo technique.

## Author contributions

**Conceptualization:** Özden Tandoğan.

**Formal analysis:** Özden Tandoğan.

**Funding acquisition:** Özden Tandoğan.

**Investigation:** Özden Tandoğan.

**Methodology:** Özden Tandoğan, Ümran Oskay.

**Resources:** Özden Tandoğan.

**Supervision:** Ümran Oskay.

**Writing – original draft:** Özden Tandoğan.

**Writing – review & editing:** Özden Tandoğan.
